# Comparative genomic analyses of the cyanobacterium, *Lyngbya aestuarii* BL J, a powerful hydrogen producer

**DOI:** 10.3389/fmicb.2013.00363

**Published:** 2013-12-11

**Authors:** Ankita Kothari, Michael Vaughn, Ferran Garcia-Pichel

**Affiliations:** ^1^School of Life Sciences, Arizona State UniversityTempe, AZ, USA; ^2^Department of Chemistry and Biochemistry, Arizona State UniversityTempe, AZ, USA

**Keywords:** biohydrogen, cyanobacteria, bidirectional hydrogenase, hoxH, hydrogen, *Lyngbya aestuarii*, microbial mats

## Abstract

The filamentous, non-heterocystous cyanobacterium *Lyngbya aestuarii* is an important contributor to marine intertidal microbial mats system worldwide. The recent isolate *L. aestuarii* BL J, is an unusually powerful hydrogen producer. Here we report a morphological, ultrastructural, and genomic characterization of this strain to set the basis for future systems studies and applications of this organism. The filaments contain circa 17 μm wide trichomes, composed of stacked disk-like short cells (2 μm long), encased in a prominent, laminated exopolysaccharide sheath. Cellular division occurs by transversal centripetal growth of cross-walls, where several rounds of division proceed simultaneously. Filament division occurs by cell self-immolation of one or groups of cells (necridial cells) at the breakage point. Short, sheath-less, motile filaments (hormogonia) are also formed. Morphologically and phylogenetically *L. aestuarii* belongs to a clade of important cyanobacteria that include members of the marine *Trichodesmiun* and *Hydrocoleum* genera, as well as terrestrial *Microcoleus vaginatus* strains, and alkalyphilic strains of *Arthrospira*. A draft genome of strain BL J was compared to those of other cyanobacteria in order to ascertain some of its ecological constraints and biotechnological potential. The genome had an average GC content of 41.1%. Of the 6.87 Mb sequenced, 6.44 Mb was present as large contigs (>10,000 bp). It contained 6515 putative protein-encoding genes, of which, 43% encode proteins of known functional role, 26% corresponded to proteins with domain or family assignments, 19.6% encode conserved hypothetical proteins, and 11.3% encode apparently unique hypothetical proteins. The strain's genome reveals its adaptations to a life of exposure to intense solar radiation and desiccation. It likely employs the storage compounds, glycogen, and cyanophycin but no polyhydroxyalkanoates, and can produce the osmolytes, trehalose, and glycine betaine. According to its genome, BL J strain also has the potential to produce a plethora of products of biotechnological interest such as Curacin A, Barbamide, Hemolysin-type calcium-binding toxin, the suncreens scytonemin, and mycosporines, as well as heptadecane and pentadecane alkanes. With respect to hydrogen production, initial comparisons of the genetic architecture and sequence of relevant genes and loci, and a comparative model of protein structure of the NiFe bidirectional hydrogenase, did not reveal conspicuous differences that could explain its unusual hydrogen producing capacity.

## Introduction

Cyanobacteria are deemed ecologically important for their contributions to global nitrogen fixation, and carbon flux (Paul, 1978; Capone et al., 1997) and their global biomass in the order of 10^14^ g C (Garcia-Pichel et al., 2003) is a relevant component of both terrestrial and marine biomes. Biotechnologically, they possess a great potential to act as cell factories by virtue of their relatively simple structure, minimal nutritional requirements, and an ability to synthesize a wide variety of metabolites. In this study, we focus on the cyanobacterium *L. aestuarii* BL J, a representative of an ecologically important species in marine intertidal mats, endowed with an extraordinary capacity to produce H_2_ (Kothari et al., 2012) and thus, of potential biotechnological interest.

In Nature, *L. aestuarii* forms extensive microbial mats in many marshes and intertidal mud flats (Horodyski and Bloeser, 1977; Mir et al., 1991; Paerl et al., 1991; Lopez-Cortes et al., 2001) Microbial mats are dense laminated benthic communities of micro-organisms (Stal and Caumette, 1993). They present an environment that is extreme in many respects, with repeated cycles of desiccation and wetting, intense exposure to ultraviolet (UV) radiation, and changing regimes of salinity (as cell may be exposed to hypersaline marine waters to very dilute meteoric precipitation). The intertidal mats that are exposed to desiccation are restricted in their anaerobic components (Rothrock and Garcia-Pichel, 2005). Although, as in most microbial communities, H_2_ is a key metabolite in interspecies metabolic linking, it rarely accumulates to concentrations high enough to be exported in significant amounts. This has been linked to the diverse populations of potential H_2_ consumers that inhabit these communities (Ebert and Brune, 1997; Schink, 1997). However, certain intertidal microbial mats, where intense net H_2_ export occurs (Skyring Gw and Smith Gd, 1989; Hoehler et al., 2001), are an exception. In an earlier report we found that, when subjected to the standard H_2_ production assays in presence of excess reductants, two different patterns were observed. The strains from marine intertidal microbial mats exhibited higher rates, steady state H_2_ concentrations and a lack of H_2_ uptake (we called this Pattern 2 H_2_ production), in comparison to those from fresh water, which exhibited lower rates and steady state H_2_ concentrations followed by uptake of most of the produced H_2_ (Pattern 1, as was known from standard strain of *Synechocytis* sp. 6803) (Kothari et al., 2012). The fresh water strain *Anabaena* sp. PCC 7120 also conformed to Pattern 1 hydrogen production. Thus, the cyanobacteria inhabiting the microbial mats (Pattern 2 H_2_ production) must have evolved extraordinarily powerful hydrogenogenic abilities to produce/sustain hydrogen under the unusually high concentrations of H_2_ prevailing in their micro-environment. Of the Pattern 2 cyanobacteria, *L. aestuarii* BL J had the highest rates and reached the highest steady state H_2_ concentrations (Kothari et al., 2012). Additionally, this strain also displayed an inducible, strong natural hydrogenogenic capacity under dark fermentative conditions (Kothari et al., in preparation). Infact, the rate of fermentative hydrogen evolution in the strain BL J was 10 times higher than that reported for the closely related strain *Oscillatoria limosa* (=*Lyngbya aestuarii* PCC 8106) (Heyer et al., 1989). Hence it was of interest to study the genome of this strain, with a special emphasis on the H_2_ producing system and the ecophysiological constraints imposed by the environment of origin.

## Materials and methods

### Strains and culture conditions

*Lyngbya aestuarii* strain BL J, a recent isolate from marine intertidal microbial mats in Baja California (Kothari et al., 2012), was grown in IMR medium set at 3% seawater salinity (Eppley et al., 1968), modified to incorporate a commercially available seawater salt mixture (Instant Ocean), instead of natural seawater, and supplemented with 0.5 μM (final concentration) NiSO_4_. The strain was maintained in axenic form on IMR media 1% agar plates (since it is less susceptible to contamination than liquid media) at room temperatures and also cryopreserved for long-term storage. Since the strain grew faster in liquid media, it was grown in 250 ml Erlenmeyer flasks, with 100 ml media in presence of 100 μmol photon m^2^s^−1^ light at room temperature to obtain cyanobacterial biomass for microscopy and DNA extractions.

### Confocal microscopy

A small pellet from liquid culture was washed and resuspended in 300 μl of fresh IMR medium. To stain the DNA, 4′, 6-diamidino-2-phenylindole, DAPI (2 μg/ml final concentration) was added. To stain the exopolysaccharide sheath, Fluorescein-labeled lectin (wheat germ agglutinin; 5 μg/ml final concentration) was added. The preparation was incubated for 1 h in dark at room temperature, and the filaments were washed thrice with fresh IMR medium. Cells were then imaged on glass slides under sealed glass coverslips using a Leica SP5 LASER scanning confocal microscope under a 63X oil immersion objective. Excitation wavelength for DAPI was at 405 nm, excitation for Fluorescein-labeled lectin was at 488 nm, and photosynthetic pigments were excited at 561 nm. The corresponding emissions were detected at 445–465, 520–535, and 675–715 nm. The images presented were maximum Z projections with corrected background (to eliminate background noise). All images were acquired at 1024 × 1024 pixel resolution. All images were manipulated using the image J software suite (Schneider et al., 2012). Imaging of hormogonia, which showed very fast gliding motility, required the use of carbonyl cyanide m-chlorophenyl hydrazine (10 μm final concentration, for 15 min, Santa Cruz biotech) as an uncoupler of proton motive force, to render them immotile.

### Transmission electron microscopy (TEM)

Unless stated all steps were under room temperature. Samples were primarily fixed in 2.5% glutaraldehyde in IMR medium for 2 h, followed by four washes in seawater medium over a period of approximately 1 h. Samples were then secondarily fixed with 1% osmium tetroxide in IMR medium for 2 h. Osmium tetroxide was removed by washing with several changes of deionized water over a period of approximately 1 h, followed by block-staining of the cells with 2% aqueous uranyl acetate for 1 h. Uranyl acetate was removed by thorough washing in deionized water. Due to poor preliminary results thought to be caused by incomplete dehydration and resin penetration of the cells, the standard TEM preparation procedure was modified to incorporate increased resident-time in dehydrating agent and epoxy resin, as well as additional gradient steps. This involved: 10 (v/v of reagent grade acetone/deionized water), 20, 40, 60, 80, and 100% anhydrous acetone for four consecutive changes, with each step lasting 30 m. A similar modified approach was employed during infiltration with Spurr's epoxy resin (Spurr, 1969): 10 (v/v of resin/anhydrous acetone), 20, 30, 50, 75%, and four consecutive changes of pure resin. Each step was under rotation for 12 h except for the 10% step, which was 3.5 h. Samples were flat-embedded in fresh resin on Teflon-spray coated glass slides and overlaid with a solid Teflon strip, then polymerized for 24 h at 60°C. Small regions of the pellet were selected and excised from the flat resin layer with a razor, then glue-mounted on a blank resin block in the desired orientation for sectioning. Ultra-thin sections (70 nm) were obtained with a Leica Ultracut-R microtome and collected on formvar-coated 1 × 2 mm slotted copper grids. Sections were post-stained for 5 m with 2% uranyl acetate in 50% ethanol solvent followed by 3 m with Sato's lead citrate (Hanaichi et al., 1986). Images were generated on a Philips CM-12 TEM operated at 80 kV and acquired by a Gatan model 791 slow-scan CCD camera.

### DNA extraction, quantification and library construction

Genomic DNA preparations were obtained by PCI (phenol; chloroform; isoamyl alcohol) extraction (Countway et al., 2005). DNA was quantified using fluorometry of ethidium bromide-stained 1% agarose electrophoresis gels and sent for commercial MiSeq 250 Illumina sequencing at the University of Maryland School of Medicine, Institute for Genome Sciences, Genome Resource Center. Genomic DNA libraries were constructed for sequencing on the Illumina platform using the NEBNext® DNA Sample Prep Master Mix Set 1 (New England Biolabs, Ipswich, MA) using the protocol provided, and after DNA fragmentation with an ultrasonicator (Covaris E210). The DNA was purified between enzymatic reactions and the size selection of the library was performed with AMPure XT beads (Beckman Coulter Genomics, Danvers, MA).

### DNA sequencing, assembly and annotation

Libraries were sequenced using the 250 bp paired-end protocol on an Illumina MiSeq sequencer. Raw data from the sequencer was processed using Illumina's RTA and CASAVA pipeline software, which includes image analysis, base calling, sequence quality scoring, and index demultiplexing. Data was then processed through both in-house pipelines for sequence assessment and quality control and FastQC (http://www.bioinformatics.bbsrc.ac.uk/projects/fastqc/). These pipelines report numerous quality metrics and perform a megablast-based contamination screen. By default, the quality control pipeline assesses basecall quality, and truncates reads where the median Phred-like quality score falls below Q20 (implying more than 99% accuracy in base calling). Data was randomly sampled to create datasets at 100, 150, and 200X coverage. The different datasets were assembled with MaSuRCA (Zimin et al., 2013). The genome assembled by MaSuRCA was subjected to the Institute of Genomic Science (Burja et al., 2001) prokaryotic annotation pipeline forms the core of the IGS Annotation Engine. The pipeline includes gene finding, protein searches, and the pFunc evidence hierarchy that produces automated functional annotation. The output of this pipeline was stored in a Chado relational database and accessed by Manatee for annotation visualization and curation (Galens et al., 2011). The genome was also annotated using RAST -Rapid Annotation using Subsystem Technology (Aziz et al., 2008).

### Genomic analyses

The genome annotated by Manatee and RAST was also analyzed manually. Homologs of certain key genes of interest were searched as queries of Psi blast of homologs from phylogenetically close protein sequences from NCBI against the entire genome. Given that the genome is not closed, there is a small probability that the genes reported as missing might be present in the unsequenced part of the genome. The Kyoto Encyclopedia of Genes and Genomes (KEGG) was employed by RAST to gain insight into the various metabolic pathway maps.

Since the bidirectional hydrogenase enzyme was central to the powerful H_2_ production exhibited by the BL J strain, the architecture of the bidirectional hydrogenase (*hox*) gene cluster, and hydrogenase accessory genes (*hyp*) was studied in detail. For the strain BL J, Manatee helped in viewing the genomic organization of the *hox* and associated ORFs. The physical map of the bidirectional hydrogenase gene cluster and associated ORFs was manually re-constructed to scale in the strains *Synechocytis* sp. 6803 and *Anabaena* sp. PCC 7120 (exemplary of Pattern 1), *Microcoleus* (=*Coleofasciculus*) *chthnoplastes* PCC 7420 and *L. aestuarii* BL J (exemplary of Pattern 2), *Lyngbya aestuarii* PCC 8106 and *Lyngbya majuscula* CCAP 1446/4 (closely related to the strain BL J; H_2_ production capacity unknown). Protein Psi Blast searches were employed to reveal if any ORFs associated with the *hox* cluster in BL J were also present in any of the other three strains.

To characterize the phylogenetic placement of this strain in reference to other strains in the same cyanobacterial subsection, 16S rRNA sequence (1322 bp) based phylogenetic tree was constructed. The sequences from 83 bacterial species were aligned using ClustalW. The alignment was manually curated and GTR (General Time Reversal model) model with GI (Gamma distributed with Invariant sites) was used to construct maximum likelihood trees with 1000 bootstrap replicates using MEGA 5.2.2 (Tamura et al., 2011).

### Bidirectional hydrogenase sequence analysis and protein modeling

The amino acid sequences of the bidirectional hydrogenase from representatives of Pattern 1 (fresh water strains: *Anabaena* sp. PCC 7120 and *Synechocystis* sp. PCC 6803) and Pattern 2 (marine intertidal strains: *M. chthonoplastes* PCC 7420 and *L. aestuarii* strain BL J) H_2_ producing cyanobacteria were used for this analysis. The protein sequences of the subunits HoxY and HoxH were individually aligned using Muscle. If the type of amino acid, changed significantly between the two Patterns but remained consistent within a Pattern, it was marked as a significant change (Supplementary Information. 1). The hydrogenase moiety (*hoxYH*) in *Synechocytis* sp. 6803 (Pattern 1) and *L. aestuarii* BL J (Pattern 2) were modeled to study the potential structural importance, of these significant amino acid positions, that might have implications for the function of the bidirectional hydrogenase. Homology models were constructed using the NiFe and NiFeSe hydrogenase templates available in the Protein Data Bank, PDB (Bernstein et al., 1977). Multiple templates were chosen from the PDB for hoxH and hoxY based on the relationship to other bacterial hydrogenases. Both subunits of 1H2A (Higuchi et al., 1997), 1E3D (Matias et al., 2001), 1FRV (Volbeda et al., 1995), 1YQW (Volbeda et al., 2005), and 1CC1 (Garcin et al., 1999) were superimposed using STAMP (Russell and Barton, 1992) and an alignment prepared based on the result using MULTISEQ (Roberts et al., 2006). Each cyanobacterial hydrogenase was profile aligned using CLUSTALW (Larkin et al., 2007) without disturbing the structure-based alignment. The PDB structure files were edited to include protein, the proximal, and medial FeS clusters, the NiFe center, CO, and CN ligands and the Fe or Mg ion at the C-terminus. The previous alignments and edited PDBs were used as inputs for MODELLER (Sali and Blundell, 1993; Eswar et al., 2002) producing 50 independent models. The resulting models were ranked by energy and those with the lowest combined energies were considered in detail. All structures were viewed and figures prepared in Visual Molecular Dynamics software, VMD (Humphrey et al., 1996). The amino acid positions changing consistently between the two patterns were highlighted to study their potential functional significance in the 3D hydrogenase model.

## Results

### Strain morphology, untrastructure, and development

**Figure 1:**
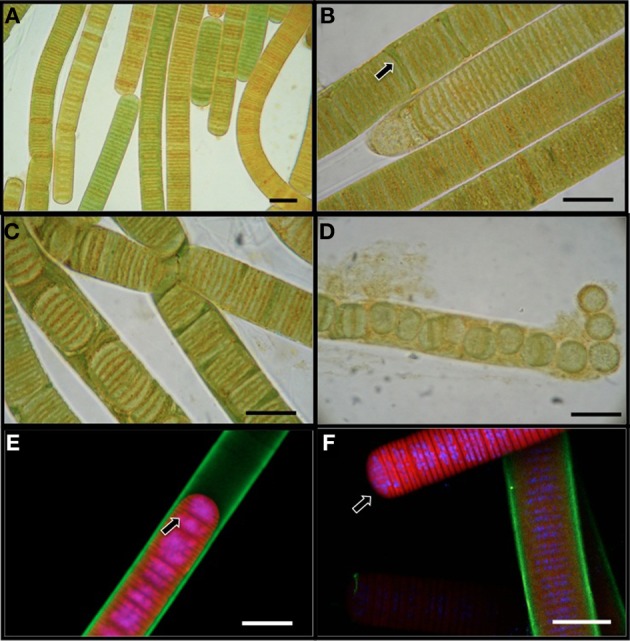
**Light microscopy images (A–D) of *L. aestuarii BL J*. (A)** The filaments display heterogeneity in pigmentation. **(B)** Formation of necridial cells (arrow). **(C)** Short filaments formed by cell division. **(D)** Breakage of trichome into individual cells or pairs of cells. Fluorescence microscopy images **(E,F)** depicting the exopolysaccharide sheath stained green, the photosynthetic pigments in red and the nucleic acids stained blue. **(E)** Cell division by transversal centripetal growth of cross-walls. Arrow marks nascent cell walls. **(F)** hormogonia (arrow) can be identified by the lack of the exopolysaccharide sheath and motility. A sheathed trichome is in the background. Bar 15 μm.

*L. aestuarii* BL J is a marine filamentous cyanobacterium belonging to the cyanobacterial subsection III according to the classification of Bergey's Manual of Systematic Bacteriology (Boone and Castenholz, 2001). The circa 17 μm wide sheathed filaments appeared in various hues of green-brown shades under the light microscope (Figure 1A), as cylindrical, unbranched, and up to 2 cm in length. The trichome consists of short disk shaped stacked cells (1.6–1.8 μm long). The cells are 14 μm wide. Confocal microscopy imaging helped to visualize the DNA and nucleiods (in blue), the exopolysaccharide sheath (in green), and the photosynthetic pigments (in red) (Figures 1E,F). A distinct mucilaginous sheath about 1.6 μm in thickness covers the trichome (Figure 1E). As evidenced by confocal microscopy, the main photosynthetic area is arranged parallel to the cross walls, and the nucleoid is central (Figure 1E). This strain often produces short, motile hormogonia as dispersal mechanisms, with little sheath (Figure 1F). The filaments develop necridic cells as a means of filament separation to make new trichomes or to aid in the formation of hormogonia (Figure 1C). Both the vegetative filaments and hormogonia have rounded terminals cells. Sometimes, single disk shaped cells were observed within the sheath and also free in the media (Figure 1D). Cell division was by formation of transversal centripetal growth of cross-walls was observed (Figure 1E), often with several consecutive rounds proceeding simultaneously. As expected, heterocysts, akinetes or any other type of specialized cells were absent.

**Figure 2:**
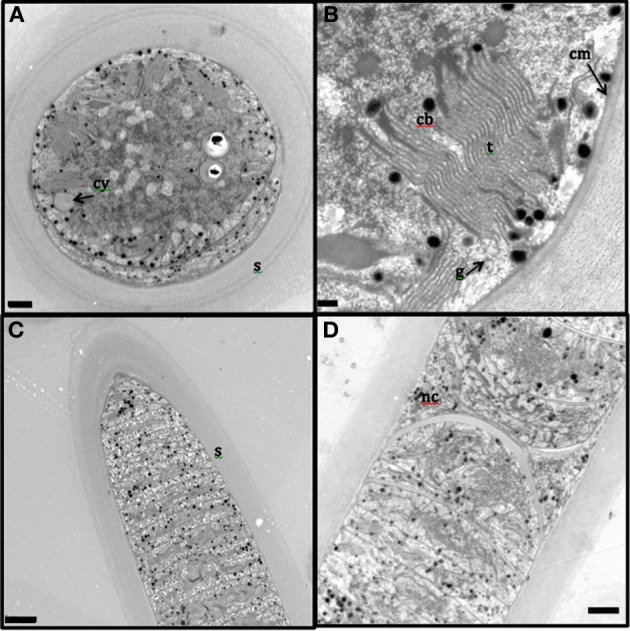
**Transmission electron microscopy images of *L. aestuarii BL J filaments***. **(A)** The transversal section with the stacked thylakoid membranes, randomly oriented, close to the periphery of the cells. The cell contains cyanophycin granules (cy) and thick sheath (s) surrounds the cell; bar 1 μm. **(B)** detailed image of the transversal section, displaying the parallel thylakoid membranes (t) along with the polyhedral carboxysomes (cb). A distinct cell membrane (cm) can be observed; bar; 0.2 μm **(C)** Longitudinal section of the filament, displaying the trichome and the thick sheath (s) around it; bar 2 μm. **(D)** Longitudinal section displaying necridial cells and newly formed trichomes within the sheath; bar 2 μm.

The presence of the thick, laminated exopolysaccharide sheath can be easily visualized in the transversal and longitudinal sections of TEM (appears wider than that observed in fluorescence microscope, perhaps, due to the TEM sample preparation, Figures 2A–D). TEM imaging revealed that the thylakoid membranes were stacked and randomly oriented and present close to the periphery of the cells (Figures 2A,B). The thylakoid membranes were associated with glycogen granules (Figure 2B). Carboxysomes (Figure 2B) and cyanophycin (Figure 2A) granules were observed in the cytoplasm. Formation of new trichomes along with necridial cells was also observed (Figure 2D).

### Whole genome sequence analysis

#### Quality of the whole genome sequence

The draft genome contained 439 contigs of which 124 were large contigs (>10,000 bp size). Based on the assembler MaSuRCA, the total contig base pair size was estimated to be 6.87 Mb. About 6.44 Mb of the entire genome was present in large contigs. The statistical measure of the median contig size or the N50 value was 80,423 Mb. This Whole Genome Shotgun project has been deposited at DDBJ/EMBL/GenBank under the accession AUZM00000000. The version described in this paper is version AUZM01000000.

#### General genomic features

The chromosome includes 6515 potential protein-encoding genes. 43% encode proteins with assigned functional role categories, 26% encode proteins with domain or family assignments, 19.6% encode conserved hypothetical proteins, which are hypothetical proteins with similarity to other hypothetical proteins and 11.3% encode hypothetical proteins, with no significant sequence similarity to other proteins. The average size of each gene was 893 bp. The tRNA and rRNA were coded by 48 and 5 genes, respectively.

In terms of whole genome DNA sequence similarity, RAST predicts that *Arthrospira maxima* strain CS-328 and *Lyngbya aestuarii* PCC 8106 (=strain CCY 9616, formerly referred to as *Oscillatoria limosa*) are the closest known strains to *L. aestuarii* BL J. Based on 16S rRNA sequence alone, the strain *L. aestuarii* PCC 8106 was the closest (99% similarity). The GC percentage of the genome *L. aestuarii* BL J was estimated to be 41.2%. This was closest to the GC percentage of *L. aestuarii* PCC 8106 (41.0%). Certain general features of the genome of sequenced strains closely related to *L. aestuarii* BL J have been tabulated (Table 1). In comparison to the closely related strains, the genome size, percent GC, predicted protein encoding genes, and total predicted genes are in the expected range.

**Table 1:** **The genome size (denoting the total contig bp sequenced for draft genomes), the percent GC, the number of protein encoding genes and the total number of predicted genes in *L. aestuarii* BL J and other closely related strains (http://www.ncbi.nlm.nih.gov/)**.

**Strain**	**Genome size (Mb)**	**Percent GC (%)**	**Protein**	**Gene**
*L. aestuarii* BL J	6.70	41.2	6515	6568
*L. aestuarii* PCC 8106	7.04	41.1	6142	6185
*Trichodesmium erythraeum* IMS101	7.75	34.1	4451	5126
*Arthrospira maxima* CS-328	6.00	44.7	5690	5728
*Arthrospira* sp. PCC 8005	6.17	44.6	5951	6094
Arthrospira platensis NIES-39	6.79	44.3	6630	6676
*Arthrospira platensis* C1	6.09	44.8	6108	6153
Arthrospira platensis str. Paraca	5.21	44.4	4674	4718
*Microcoleus vaginatus* FGP-2	6.70	46.0	–	–
*Microcoleus chthonoplastes* PCC 7420	8.68	45.4	8294	8347
*Microcoleus* sp. PCC 7113	7.97	46.2	6441	6821

“–” Data unavailable on the NCBI website.

#### Energy metabolism

As expected, this strain had the homologs of the complete sets of genes coding for both photosystem I, (14 genes; some with additional homologs) and photosystem II, (22 genes; some with additional homologs). It also had homologs of genes coding for phycobilisome proteins, phycocyanin, and allophycocyanin. We did not detect homologs of genes coding for phycoerythrocyanin and phycoerythrin. Complete set of genes required for the Calvin cycle (12 genes; some with additional homologs) along with the presence of key enzymes RuBisCo (RbcL), phosphoribulokinase (PRK), and sedoheptulose-1,7-bisphosphatase (SBP) were present, as were the essential genes involved in the Carbon-dioxide Concentrating Mechanism, or CCM (15 genes; some with additional homologs). The gene *hat/hatR* (high affinity carbon uptake protein) has 31 homologs in BL J. In strains *L. aestuarii* PCC 8106, *M. chthonoplastes* PCC 7420, and *Acaryochloris marina*, similarly high number of homologs of the same gene can be found, while other strains like *Prochlorococcus marinus* MIT 9215, *Synechococcus* sp. WH 8102, *Synechococcus* sp. CC9311, *Cyanothece* sp. PCC 8801, and *Synechocystis* sp. PCC 6803, just contain none to two at most.

Homologs of genes coding photoprotective proteins such as flavodiiron proteins (FPD's) and orange carotenoid proteins were also present. The genome of *Synechocystis* sp. PCC 6803 contains 4 putative flavodiiron protein-coding genes (CyanoBase: http://bacteria.kazusa.or.jp/cyanobase/) of which two (*sll0219* and *sll0217*) have a role in photoprotection of the cells and in the sustenance of the photosystem II (PSII) complex (Zhang et al., 2009). In comparison, the strain BL J has hosts only 2 putative flavodiiron protein coding genes and with no corresponding homolog to the gene *sll0219* in *Synechocystis* sp. PCC 6803. It has the homologs of genes coding for the photoprotective orange carotenoid protein, also present in closely related strains such as *A. maxima* CS-328 and *L. aestuarii* PCC 8106.

With respect to dark carbon catabolic metabolism, it has the homologs of all genes required for glycolysis, Entner-Doudoroff pathway and the pyruvate pentose phosphate pathway. The TCA cycle has homologs of genes similar to that reported in other cyanobacteria, including those (namely 2-oxoglutarate decarboxylase and succinic semialdehyde dehydrogenase) reported to be involved in a cyanobacterial type of TCA cycle (Zhang and Bryant, 2011). The ortholog of the gene coding for succinic semialdehyde dehydrogenase in the strain BL J is reduced (59% query coverage) compared to that observed in *Synechococcus* sp. PCC 7002. Similar results were observed in the orthologs of the same gene in closely related strains such as *L. aestuarii* PCC 8106 (68% query coverage) and *Trichodesmium erythraeum* IMS101 (64% query coverage) when compared to the succinic semialdehyde dehydrogenase gene observed in *Synechococcus* sp. PCC 7002.

This strain has all the genes required for mixed acid fermentation (8 genes) for surviving through dark anaerobic conditions [which, in fact, it carries out; (Kothari et al., in preparation)]. Even though a capacity for anoxygenic photosynthesis is typical from microbial mat cyanobacteria (Garcia-Pichel and Castenholz, 1990) we could not detect homologs of the enzyme sulfide:quinone oxidoreductase that catalyses the initial step in sulfide-dependent donation of electrons to PSI.

#### Nitrogen metabolism

Cyanobacteria have the ability to use various organic and inorganic sources of nitrogen from the environment. (Luque and Forchhammer, 2008). BL J has all the homologs required for fixing atmospheric nitrogen into ammonium. This includes the structural genes *nifD* and *nifK* encoding the dinitrogenase moiety and *nifH* encoding the dinitrogenase reductase. As observed earlier in filamentous strains like *Anabaena* sp. PCC 7120 (Haselkorn et al., 1998) and *L. aestuarii* PCC 8106 the genes *nifBSUHDKENX* are clustered and oriented in a single direction. The homologs of genes coding for the uptake hydrogenase enzyme involved in consumption of the H_2_ produced by the nitrogenase, are also present in this strain. The genes of coding for the uptake hydrogenase (*hupSLW*) are generally clustered and oriented in the same direction. In this strain *hupW*, the putative C terminal endopeptidase, lies several kb downstream of the main locus. Interestingly, this strain possesses homologs of the gene *hetR* involved in the formation of heterocysts (Buikema and Haselkorn, 1991), even though it does not develop heterocysts. All genes required for reducing inorganic nitrate into ammonium, including nitrate reductase (*nar*) and nitrite reductase (Panda et al., 2008), are present. This strain also hosts homologs of genes corresponding to uptake of organic sources of nitrogen (urea) and amino acids (see below), and a homolog of the urease gene.

Ammonium ion assimilation constitutes a central metabolic pathway in cyanobacteria wherein Glutamine synthetase (Burja et al., 2001) and an NADPH-dependent glutamine 2-oxoglutarate amidotransferase (GOGAT) plays the primary role of ammonium ion incorporation into glutamine and glutamate (Muro-Pastor et al., 2005). This strain has the homologs of both Glutamine synthetase and a NADPH-dependent GOGAT. The homolog of the gene coding for *ntcA* (Vega-Palas et al., 1992) involved in global nitrogen control is also present in this strain.

#### Signal transduction

The sensory kinases (involved in sensing the environmental changes) and the response regulators (involved in regulating gene expression) together constitute the “two-component system.” This signal transduction system aids bacteria in adapting to their environmental changes. Only the orthologs of genes coding for the classic two-component systems were detected. This strain has 100 genes coding for the two-component systems (similar values reported in other strains). Of the 100 genes 42 encode histidine kinase A domain protein and the rest code for response regulators.

Additionally, 57 other ORFs were detected with putative role in signal transduction. Of these, about 51 ORFs were assigned as the diguanylate cyclase domain protein-coding gene which participates in the formation of the ubiquitous second messenger cyclic-di-GMP (Ross et al., 1987). The other six ORFs were assigned to the EAL domain protein-coding gene, which is associated with the diguanylate cyclase protein domain. It is a conserved protein domain, proposed to function as diguanylate phosphodiesterase (Galperin et al., 2001).

#### Transport and binding proteins

This strain has multiple ORFs with predicted function as binding protein-dependent transport systems. 590 ORFs are predicted to have a role in coding for transport and binding proteins for amino acids, peptides, and amines (9), anions (24), carbohydrates, organic alcohols, and acids (15), cation (56), nucleosides, purines, and pyrimidines (2), Porins (4), other substrates such as heme or polysaccharides (17) and unknown substrate (463). The ORFs involved in anion binding and transfers were homologs of ABC transporter coding genes proposed to transfer phosphate, sulfate, nitrite, phosphonate, phosphite, and molybdate. The ORFs involved in cation binding and transfer were proposed to transport the cations: sodium, copper, ferrous, cadmium, cobalt, magnesium, calcium, potassium, and nickel.

#### Organic osmotic solutes

The solute trehalose is characteristic of low-salt tolerant cyanobacteria (Oren et al., 1994), such as *Scytonema* sp. (Page-Sharp et al., 1999), *Anabaena* sp. PCC 7120 (Higo et al., 2006). But it is also present in some marine cyanobacteria such as *Crocosphaera watsonii* WH8501 (Pade et al., 2012). The genome of this strain had homologs of the enzymes trehalose synthase and trehalose-6-phosphate synthetase involved in trehalose synthesis. However, we did not detect the enzyme trehalase involved in its breakdown. *L. aestuarii* PCC 8106, seems to use trehalose as a storage compound (Heyer et al., 1989). Glucosylglycerol is an osmolyte commonly seen in moderately halotolerant cyanobacteria *Synechocystis* PCC 6803 (Hagemann and Erdmann, 1997), *Arthrospira* (=*Spirulina*) *platensis* (Warr et al., 1985), *Synechococcus* sp. strain 7002 (=*Agmenellum quadruplicatum* PR6) (Tel-or et al., 1986), *Microcystis firma* strain Gromow 398 (Erdmann et al., 1992), and *Oscillatoria* sp. SAG 3192 (Moezelaar et al., 1996). However, we did not detect the genes required for the synthesis of glucosylglycerol in this strain. Glycine betaine is charachteristic of highly halotolerant cyanobacteria *Halothece* (*Aphanothece) halophytica* (Reed et al., 1984), *Halospirulina tapeticola* (Nubel et al., 2000), *Spirulina subsalsa* (Gabbay-Azaria et al., 1988), *Halothece* (*Dactylococcopsis) salina* (Moore et al., 1987), and *Synechocystis* sp. DUN52 (Mohammad et al., 1983). This solute has also been previously detected in *Oscillatoria* mats (Oren et al., 1994) inhabiting the hypersaline sulfur hot springs at Hamei Mazor. In cyanobacteria, the enzymes choline dehydrogenase and betaine aldehyde dehydrogenase catalyze the formation of this osmolyte (Oren et al., 1994). Homologs of both of these genes were present in the strain BL J indicating that this strain has the genetic capacity to make glycine betaine.

#### Storage compounds

Glycogen is a major carbohydrate reserve molecule in cyanobacteria. Homologs of all the genes involved in glycogen metabolism were detected. Elsewhere we showed that glycogen is stored in the light and mobilized in the dark either aerobically or anaerobically (Kothari et al., in preparation). Cyanophycin (multi-L-arginyl-poly-L-aspartate) is a water-insoluble, high nitrogen reserve polymer (Ziegler et al., 1998), quite commonly encountered as a carbon and nitrogen storage polymer in cyanobacteria. (Huang and Chou, 1991; De Philippis et al., 1992; Miller and Espie, 1994). Homologs for the cyanophycin synthetase were also found in BL J. 2 homologs of the cyanophycinase, a peptidase degrading cyanophycin were present, one of which followed the cyanophycin synthetase gene. Polyhydroxyalkanoates or PHAs are linear polyesters storage carbon and energy compounds seen in many cyanobacteria (Stal, 1992; Asada et al., 1999; Hai et al., 2001; Panda et al., 2008; Shrivastav et al., 2010). However, this strain seems to lack the homologs for poly (3-hydroxyalkanoate) synthase (*phaC*), the key enzyme for PHA synthesis.

#### Genes of biotechnological importance

*Secondary metabolites:* Polyketide synthases (PKSs) are a family of multi-domain enzymes that produce polyketides, a large class of secondary metabolites. The strain *L. aestuarii* BL J has genes homologs to the putative polyketide synthase module-related protein PKS in *Moorea producens* 3L (Jones et al., 2011). Homologs of genes involved in Curacin A and Barbamide synthesis (Jones et al., 2011) were also present as were homologs of genes coding for Hemolysin-type calcium-binding toxin seen in *L. aestuarii* PCC 8106. 3 putative homologs of genes coding for the putative RTX toxin (a type of cytotoxin) along with toxin secretion ABC transporter ATP-binding protein seen in *L. aestuarii* PCC 8106 were detected as well. Certain cyanobacteria synthesize a protective pigments in response to UV irradiation (Karsten et al., 1998; Gao and Garcia-Pichel, 2011b). Among them, scytonemin, has been directly detected in *L. aestuarii* mats (Garcia-Pichel and Castenholz, 1991). Previously it has been reported that the scytonemin genes *scyABCDEF* are clustered with all the genes oriented in the same direction in a few cyanobacterial strains (Soule et al., 2009). Similar arrangement of *scy* genes was observed in this strain. Homologs of all the genes essential for the biosynthesis of tryptophan from chorismate (*trpE, trpC, trpA, trpB, trpD*) are present and they are oriented in the direction opposite to that of the scy gene cluster. In fact, the scytonemin gene cluster in the strain BL J is exactly the same as seen in *L. aestuarii* PCC 8106 (Soule et al., 2009). In response to UV-B irradiation, certain cyanobacteria synthesize mycosporines. This strain has the homologs of all the 3 genes involved in the formation of mycosporine-glycine from sedoheptulose-7-phosphate. All these 3 genes are clustered and oriented in the same direction as reported in *Anabaena variabilis* ATCC 29413 and *Nostoc punctiforme* ATCC 29133. There is a homolog of the gene involved in the conversion Mycosporine-glycine to Shinorine, present elsewhere in the genome (Gao and Garcia-Pichel, 2011a).

Certain cyanobacterial strains (Schirmer et al., 2010; Starkenburg et al., 2011) have the genes coding for the synthesis of heptadecane and pentadecane alkanes; a major constituent of gasoline, diesel, and jet fuel. The homologs of genes acyl-[acyl carrier protein] (ACP) reductase and aldehyde decarbonylase involved in the synthesis of heptadecane and pentadecane alkanes (Schirmer et al., 2010) are present in this strain. Homologs of these genes are also present in closely related strains such as *L. aestuarii* PCC 8106, *Microcoleus vaginatus* FGP-2, and *Trichodesmium erythraeum* IMS101.

#### Other genes of interest

This strain has genes for resistance to copper, cobalt, zinc, cadmium, mercury, fluoroquinolones, arsenic, and beta-lactam antibiotics. The homolog of the gene involved in biotin synthesis (*BioA*) could not be detected in this strain. However, it did encode genes for biotin uptake from the environment (and biotin was a constituent of the IMR medium), a trait also seen in other closely related strains. The genome has genes coding for *hipBA*, which are proposed to have a role in formation of persister cells (dormant cells with antimicrobial resistance) in response to antibiotic and other stresses (Jayaraman, 2008). The genome has genes corresponding to Internalin- putative, *Inl*, and Internalin A, *InlA* that are implicated internalization or virulence in *Listeria* (Cossart and Lecuit, 1998).

### Comparative analysis of the bidirectional hydrogenase and accessory proteins

#### Bidirectional hydrogenase

**Figure 3:**
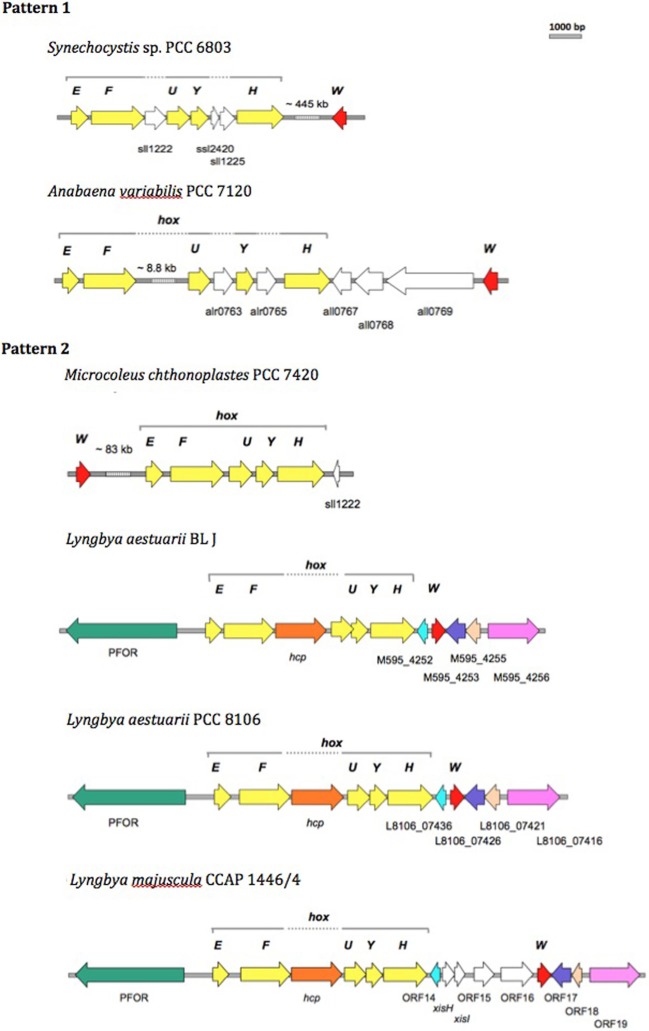
**Comparison of the physical map of the bidirectional hydrogenase gene cluster and associated ORFs in Pattern 1 (*Synechocytis* sp. 6803 and *Anabaena* PCC 7120) and Pattern 2 (*M. chthnoplastes* PCC 7420 and *L*. *aestuarii* BL J) H_2_ production displaying strains**. The genomic regions from other closely related *Lyngbya* species (*L. aestuarii* PCC 8106 and *L. majuscula* CCAP 1446/4) are included for reference (Ferreira, 2009). The following ORFs are depicted: *hox* genes (yellow ORFs), *hoxW* (red ORFs), and some additional ORFs (shown as white ORFs, or colored ORFs (including Pyruvate Formate Oxido Reductase, PFOR; Hybrid Cluster Protein, hcp) when homologs to the ones in *L. aestuarii* BL J).

We compared the bidirectional hydrogenase and hydrogenase accessory gene cluster of strains displaying either Pattern 1 (*Synechocystis* sp. PCC 6803 and *Anabaena* sp. PCC 7120) or Pattern 2 (*L*. *aestuarii* BL J and *M. chthonoplastes* PCC 7420) H_2_ production, and included comparisons with closely related *Lyngbya* species, *L. aestuarii* PCC 8106, and *L. majuscula* CCAP 1446/4 (of untested H_2_ production capacity). *L. aestuarii* strain BL J hosts a Ni-Fe bidirectional hydrogenase enzyme the locus of which is a 6.89 kb gene cluster. The genes coding for the bidirectional hydrogenase (*hoxEFUYH*) are often grouped together as in the strain PCC 7420, but a few other ORFs are interspersed in the cluster of *Synechocystis* sp. PCC 6803 (Schmitz et al., 1995). In *Synechococcus elongatus* and in *Anabaena* sp. PCC 7120 (Boison et al., 1998; Kaneko et al., 2001) the two clusters, *hoxEF*, and *hoxUYH* are separated by several kb. The clusters *hoxEF and hoxUYH are* separated by a single gene coding for *hcp* (encoding a putative hybrid cluster protein) in *Lyngbya* strains CCAP 1446/4, PCC 8106 (Ferreira, 2009), and BL J. In fact, the overall arrangement of genes and ORFs in the hydrogenase cluster in *L. aestuarii* BL J is undistinguishable from that seen in PCC 8106 (Figure 3).

In terms of protein sequence, the bidirectional hydrogenase from *L. aestuarii* PCC 8106 was the closest to that of *L. aestuarii* BL J. Identities in the hoxE, F, U, Y and H between the two strains were 97, 97, 97, 96, and 95%, respectively, and gene lengths for each subunit were the same in both strains. Similar to what was previously reported for *L. majuscula* CCAP 1446/4 and *L. aestuarii* PCC 8106 (Ferreira, 2009), the ORF before *hoxE* is annotated as a pyruvate ferredoxin oxidoreductase in the strain BL J. Pyruvate ferredoxin oxidoreductase is a key enzyme in fermentation, and is typically active in dark anaerobic conditions along with the bidirectional hydrogenase (Kletzin and Adams, 1996). Homologs of the ORF M595_4252 (belonging to BL J strain) are also found in PCC 8106 (L8106_07436) and CCAP 1446/4 (ORF 14) strain. This ORF codes for a hypothetical protein with three predicted transmembrane helices along with homology to cyanobacterial genes coding for putative membrane proteins. Similarly, homologs of other hydrogenase-cluster-associated-ORFs in BL J strain (M595_4253, M595_4255, and M595_4256) were seen in PCC 8106 and CCAP 1446/4 strains (Figure 3).

The gene, *hoxW*, codes for a carboxyl-terminal protease that releases a 24-amino-acid peptide from HoxH prior to progression of subunit assembly (Thiemermann et al., 1996). *HoxW* is found immediately downstream of *hoxH* in *S. elongatus* PCC 6301, *S. elongatus* PCC 7942, and *Synechococcus* sp. PCC 7002. However, *hoxH* and *hoxW* are separated by several kb in *Synechocystis* sp. PCC 6803 (Kaneko and Tabata, 1997) and *M. chthonoplastes* PCC 7420. Three ORFs separate the *hoxH* and *hoxW* in *Anabaena* sp. PCC 7120. A single ORF separates the *hoxH* gene from *hoxW* in the *L*. *aestuarii* strains (*L. majuscula* CCAP 1446/4, *L*. *aestuarii* PCC 8106, and *L*. *aestuarii* BL J).

The genes *hypFCDEAB* code for the maturation of bidirectional hydrogenase in cyanobacteria (Lutz et al., 1991; Jacobi et al., 1992). Amongst the Pattern 1 exhibiting strains, the *hyp* genes are dispersed in the genome of *Synechocystis* sp. PCC 6803 (Kaneko et al., 1996) whereas they are clustered and oriented in the same direction in *Anabaena* PCC 7120 with an additional ORF (coding for probable 4-oxalocrotonate tautomerase) between the *hypD* and *hypE* genes. In PCC 7420, one finds two clusters (*hypAB* and *hypFCDE*) with additional hypothetical ORFs between *hypF* and *hypC* and another one between *hypD* and *hypE*. In the strain BL J, *hypFCDEAB* are clustered, with all genes oriented in the same direction and encompassing two additional ORFs coding for hypothetical proteins. A similar arrangement has been observed in *L. aestuarii* PCC 8106. In BL J, *hypC* has one additional homolog in the genome, as does *hypF*, but the latter is highly truncated (13% query coverage). No additional homologs of *hyp* genes are found in *Lyngbya* strains (CCAP 1446/4 and PCC 8106). In *Synechocystis* PCC 6803, additional homologs hypA2 and hypB2 were clustered but they don't seem to play a role in maturation of the bidirectional hydrogenase (Hoffmann et al., 2006). Homologs of the gene *hypX*, with a proposed role in oxygen tolerance of soluble Ni-Fe hydrogenases in *Ralstonia eutropha* H16 (Bleijlevens et al., 2004), could not be detected in the strain BL J. Thus, at the level of the physical map of the bidirectional hydrogenase and accessory gene cluster we did not observe any congruent changes consistent within a hydrogen production Pattern.

#### Protein modeling of the bidirectional hydrogenase

We modeled the 3D structure of the hydrogenase subunit of the bidirectional hydrogenase from *L. aestuarii* BL J (as an exemplary of Pattern 2) and *Synechocystis* sp. PCC 6803 (as an exemplary of Pattern 1) based on the genomic sequence. The model was constructed based on five related bacterial NiFe hydrogenases from the Protein Database. In general, the overall fold and length of the large subunit, hoxH, was similar to the heavy chain of the bacterial NiFe hydrogenase model templates. The C-terminus of the NiFe hydrogenase aids in nickel insertion prior to its cleavage to allow a structural reorganization of the whole molecule, and the consequent assembly of the holoenzyme (Fritsche et al., 1999). It has been suggested that in the cyanobacterial bidirectional hydrogenases, the last 25–32 C-terminal amino acids are cleaved (Tamagnini et al., 2007). The alignments leading to the homology models presented in this work demonstrate strong homology in the C terminal region and both the strains are consistent with an excised C-terminal portion of 25 amino acids. As reported earlier, the small subunit, hoxY, is significantly shorter in the cyanobacterial bidirectional hydrogenases than in the light chain of the bacterial NiFe hydrogenase model templates. The light chain template structures of NiFe hydrogenases that corresponded to hoxY consisted of 2-folded domains connected through a linear unstructured sequence. Only the first domain of the light chain template is homologs to the cyanobacterial hoxY sequence. The amino acids corresponding to hoxY only house one of the three FeS, which corresponds to the proximal FeS cluster observed in the light chain of the bacterial NiFe hydrogenase model templates.

**Figure 4:**
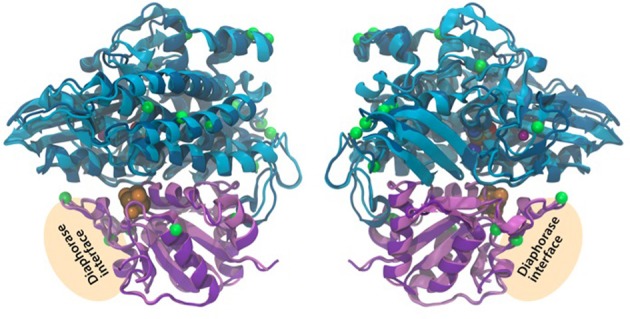
**Three-dimensional homology model of the hoxYH subunits from *L. aestuarii* BL J and *Synechocystis* sp. PCC 6803**. The backbone of each protein is depicted in ribbons with *Synechocystis* in dark blue (hoxH) and dark purple (hoxY) and *L. aestaurii* in light blue (hoxH) and light purple (hoxY). The images are related by a 180° rotation of the model along the Y-axis. The positions of the amino acids that vary significantly between the pattern 1 and pattern 2 have their alpha carbon depicted in space-filling green. The proposed diaphorase interface is also depicted in the figure. The cofactors shown at their van der Waals radius and are colored as follows: Orange, sulfur; brown, iron; blue, nickel; cyan, carbon; red, oxygen; and maroon, magnesium.

The 2-fold purpose of the structural comparison was to determine if there were significant structural differences in the enzyme itself and if the consistent amino acids substitutions between representatives of the two hydrogen production Patterns, were located in proximity to active sites in the enzyme. Our results indicate that the overall fold and domain structures between the hydrogenase subunits of *L. aestuarii* BL J and *Synechocystis* sp. PCC 6803 were indeed very similar (Figure 4). In comparing protein sequences we found that there were 15 positions in hoxH and 5 in hoxY where the type of amino acid remained consistent within a Pattern but varied amongst the two Patterns (see supplementary information 2). However, all of these significant amino acid changes between the two Patterns lie on the exterior loops in the model (Figure 4) indicating they were not crucial in explaining the differences in the H_2_ producing physiologies observed between the two Patterns.

## Discussion

Our genomic and cellular description of *L. aestuarii* BL J shows that this strain shares phylogenetic placement, morphological, and life history traits, with certain other environmentally and biotechnologically relevant cyanobacteria. This clade (Supplementary Information. 2) encompasses globally important marine cyanobacteria like *Trichodesmium* spp. and globally relevant terrestrial forms such as *Microcoleus vaginatus*, estimated to be the 3rd and 4th most abundant cyanobacteria on the planet, respectively (Garcia-Pichel et al., 2003). The group includes strains in the genus *Arthrospira* of importance for large scale production of biomass (Hu, 2004) and used commercially as “*Spirulina*” as a health food additive (Milledge, 2011) and, of course many other strains of *Lyngbya* in the so-called Halophilic/brackish/freshwater cluster of biotechnological fame because of their rich and diverse set of secondary metabolites (Engene et al., 2011). All of them being filamentous, non-heterocystous, with discoidal, rather large cells that undergo several rounds of division simultaneously by invagination of cross-walls, and that develop necridial cells for the formation of dispersive hormogonia and for filament separation (Figures 1, 2). Unfortunately given their importance, there are currently no members of this clade for which a system of genetic manipulation has been developed, heavily curtailing biotechnological advancement and bringing and added value to genomic investigations of their members. Given this lack, genomics provides an opportunity to identify and transform genes of interest into other model organisms.

The strain *L. aestuarii* PCC 8106, isolated from a similar intertidal habitat (a microbial mat in the German Wadden Sea island of Mellum), was the closest to BL J both in terms of the 16S rRNA sequence similarity and phylogeny, and many of the other genomic features (the genome size, percent GC, predicted protein encoding genes and total predicted genes; Table 1). However, significant differences exist between these two strains. Perhaps the most conspicuous being that BL J is almost 50% larger than PCC 8106, with reported cell width around 10 μm (Stal and Krumbein, 1981). Other features like the arrangement of the thylakoid membranes in stacks does not occur in PCC 8196 either (Stal and Krumbein, 1981). We also report the occasional presence of loose, disk-shaped cells within the sheath and the in the media, indicating that cell-to-cell linkages in our strain can be weak, in what can potentially be relevant as an additional means of dispersal that will require focused study.

Our genomic predictions found confirmation in a variety of traits that could be independently assessed. For example, electron microscopy (Figure 2) confirmed the predicted presence of glycogen as a major carbohydrate storage molecule in this strain to the exclusion of polyhydroxyalkanoates. It did also confirm the genomic predictions of cyanophycin synthesis (Figure 2A) and the formation of carboxysomes. Light microscopy revealed the presence of scytonemin in the sheaths of BL J (and we could confirm its preferential synthesis under added UV-A radiation; data not shown), supporting the finding of the entire scytonemin operon in the genome. This all lends credence to other yet to be supported predictions.

A reading of *L. aestuarii* BL J's genome also speaks directly to some of the environmental constraints of this species in its environment of origin. Known to inhabit exposed intertidal surfaces and the topmost layers of the microbial mats, a high-light phenotype can clearly be surmised from the presence of many photoprotective mechanisms, from extra and intracellular sunscreens, to FPD's that regenerate excess electrons by reducing molecular oxygen to water (Goncalves et al., 2011), to orange carotenoid protein, which helps decouple the light-harvesting systems from the reaction centers (Wilson et al., 2006), as well as from the absence of genes coding for light harvesting pigments that can be considered adaptations to low light intensity like phycoerythrin (Kana and Glibert, 1987), phycoerythrocyanin (Prufert-Bebout and Garcia-Pichel, 1994), or chlorophyll d (Swingley et al., 2008). Intertidal habitats are recurrently exposed to cycles of desiccation and rewetting. Not much is known about the genes involved in desiccation resistance in cyanobacteria but recent transcriptomic studies on the terrestrial strain *Microcoleus vaginatus* indicate than this is a complex response that involves large sets of genes (Rajeev et al., 2013) and which include complex DNA repair responses, up-regulation of reactive oxygen detoxification mechanisms, the production of osmolites and upregulation of orange carotenoid proteins. Many of the genes involved in these adaptations are also present in this strain, but its mere presence cannot necessarily be linked to desiccation stress resistance. This strain has clearly acquired mechanisms to hold on to moisture, however. In fact, it was very difficult to dehydrate the filaments of this strain for the purpose of TEM preparation. Its thick sheath and the predicted presence of glycine betaine, unusual for a non-halophilic strain, in addition to trehalose, might help in providing desiccation resistance. Finally, a condition typical of the mat habit is that diffusion becomes the major transport mechanism for substrates and products of metabolism. This tends to create diffusion limitations to metabolic activities like photosynthesis and respiration (Garcia-Pichel et al., 1994), which gives relevance to the presence of homologs of genes coding for high affinity carbon uptake protein (*hat*) and carbon concentrating mechanism (*ccm*) along with abundant carboxysomes (Figure 2). It also promotes the establishment of anaerobiosis at night within the mat due to consumption by respiration. Under these conditions fermentation of internal reserves though a mixed acid pathway is the only energy-generating metabolism available to strain BL J. Interestingly, this strain lacks the capacity to perform anoxygenic photosynthesis using hydrogen sulfide as an electron donor (homologs of the gene coding for sulfide quinone oxidoreductase were missing) common in microbial mat cyanobacteria. Perhaps this is linked to the low concentrations of sulfide in the upper layers of these intertidal mats compared to mats that are constantly submerged, and where *Lyngbya* never dominates (Rothrock and Garcia-Pichel, 2005). The presence of recurrent anaerobic conditions will also make soluble ferrous iron available, perhaps leading to the fact that adaptations to iron deficiency such as the products of “iron-stress-induced” gene, *isiA*; (Straus, 1994; Park et al., 1999) were not detected in BL J.

On the biotechnological potential of this strain we have to note its apparently very rich set of secondary metabolites that range from toxins like Curacin A, Barbamide, Hemolysin-type calcium-binding toxin, to suncreens like scytonemin and mycosporines, to biofuel prospects like heptadecane and pentadecane alkanes. But clearly, biohydrogen is the most promising product of biotechnological importance from this strain (Kothari et al., 2012). Since the standard assays for hydrogen production were performed in the presence of nitrate, a condition in which nitrogenase is not known to be inactive (Ferreira et al., 2009), we rule out the role of nitrogenase in the production of hydrogen. The uptake hydrogenases are known to produce little hydrogen in presence of reduced methyl viologen (Houchins and Burris, 1981). In contrast, reduced methyl viologen is commonly used to assay the bidirectional hydrogenase activity and is likely the enzyme majorly contributing to the strong hydrogen producing capacity of the strain BL J described previously (Kothari et al., 2012). Therefore, the bidirectional hydrogenase gene cluster in this strain is studied in detail with comparisons drawn to other hydrogen producing strains. The organization of the bidirectional hydrogenase (*hox*) and accessory hydrogenase (*hyp*) gene cluster was unique in all the four strains (*Synechocystis* sp. PCC 6803, *Anabaena* sp. PCC 7120, *L*. *aestuarii* BL J, and *M. chthonoplastes* PCC 7420). *A priori*, the comparative analysis of the organization of the bidirectional hydrogenase and accessory genes locus revealed no major changes consistent within a Pattern but varying between the two Patterns. A comparative analysis of the organization of the bidirectional hydrogenase locus in the strain BL J revealed that it was similar to that of *L. aestuarii* PCC 8106 and showed only minor differences with that of *L. majuscula* CCAP 1446/4 strain (Figure 3).

Interestingly, in all of the *Lyngbya* strains (Figure 3), a homolog of the *hcp* gene, predicted to code for hydroxylamine reductase (Wolfe et al., 2002) and typically associated with detoxification of by-products of nitrate reduction (Cabello et al., 2004), is found between genes hoxF and hoxU. Interestingly, in cyanobacteria that are strong hydrogen producers and display sustained concentrations of hydrogen for more than 24 h in dark anaerobic conditions (Ananyev et al., 2008; Kothari et al., in preparation), the *hcp* gene is present, while it is absent from the genomes of Pattern 1 strains, namely, *Synechocystis* sp. PCC 6803 and *Anabaena* sp. PCC 7120. This coincidence may provide a hypothesis worth elucidating the high hydrogenogenic capacity of the Pattern 2 strains. Although typically hcp is annotated as hydroxylamine reductases, it also presents significant homology to known carbon monoxide dehydrogenase (CODH). Perhaps the product of hcp plays a role in the generation of CO needed (Pierik et al., 1999) for the maturation of the NiFe hydrogenases. The source of the CO ligand in the NiFe hydrogenases continues to be unknown (Bürstel et al., 2011).

At the level of hoxYH sequence comparison, we could detect some amino acids substitutions that were consistent within a Pattern but differed amongst the two Patterns. However, none of these amino acids mapped close to the enzyme's active sites, when located on 3D structural models of the hydrogenases of *L. aestuarii* BL J or *Synechocystis* PCC 6803 (Figure 4), implying that they are unlikely to modify reaction rates. This suggests that polypeptide differences of the hydrogenase enzyme between the two Patterns are unlikely to explain the functional differences detected previously, necessitating, further study of the biochemistry and regulation of the bidirectional hydrogenase enzymes in these strains. Heterologous expression of the bidirectional hydrogenase from *L. aestuarii* BL J in model strains such as *Synechocystis* PCC 6803 might help in gaining a better understanding of the enzyme system.

## Author contributions

Concept by Ferran Garcia-Pichel and Ankita Kothari, experimental work (phenotypic and genetic analyses) by Ankita Kothari and 3D hydrogenase enzyme modeling by Michael Vaughn. Writing by Ankita Kothari (with assistance from Michael Vaughn on 3D enzyme modeling) and editorial help by Ferran Garcia-Pichel.

### Conflict of interest statement

The authors declare that the research was conducted in the absence of any commercial or financial relationships that could be construed as a potential conflict of interest.

## Abbreviations

DDBJ: DNA Database of Japan
EMBL: European Molecular Biology Laboratory.
