# Supplementary material for: Comparative genomic analyses of the cyanobacterium, Lyngbya aestuarii BL J, a powerful hydrogen producer
Source: Front Microbiol. 2013 Dec 11;4:363. doi: 10.3389/fmicb.2013.00363 (PMC3858816; doi:10.3389/fmicb.2013.00363)
Supplement: Supplementary file 1 [file DataSheet1.DOCX]

Supplementary Information 1. Maximum likelihood tree based on the 16S rRNA sequence of 76 bacterial strains. Bootstrap values (%) are indicated, obtained from analyses of 1000 replicate trees. *Microcoleus chthonoplastes* cluster consists of strains WW2, CCY9608, NDN, 7420 and CHI; Heterocystous cluster contains strains *Scytonema* sp. IAM M26, *Scytonema* sp. u-3-3, *Scytonema hofmanni* PCC 7110, *Calothrix* sp. XP9A, *Rivularia* sp. XP 16B, *Rivularia* sp. PCC 7116, *Calothrix deserica* PCC7102, *Rivularia* IAM M26 1, *Calothrix* sp. PCC 7714, *Mastigocladus laminosus* Ja, *Nodularia* *spumigena* AV63, *Nodularia spumigena* PCC 9350, *Nodularia sphaerocarpa* PCC 7804, *Nostoc* sp. PCC 7120, *Anabaena variabilis* ATCC 29413, *Tolypothrix* sp. PCC 7415, *Tolypothrix* sp. PCC 7504, *Tolypothrix* sp. PCC 7101; *Synechococcus* cluster contains strains PCC 7310, 7117, 7002 and the *Euhalothece* cluster contains strains MPI95AH10, MPI96N303, MPI96N304.
