# Supplementary material for: Comparative genomic analyses of the cyanobacterium, Lyngbya aestuarii BL J, a powerful hydrogen producer
Source: Front Microbiol. 2013 Dec 11;4:363. doi: 10.3389/fmicb.2013.00363 (PMC3858816; doi:10.3389/fmicb.2013.00363)
Supplement: Supplementary file 2 [file DataSheet2.DOCX]

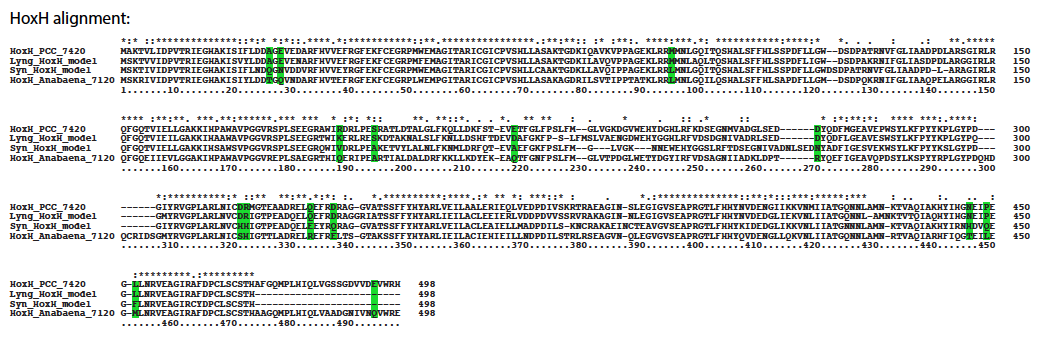


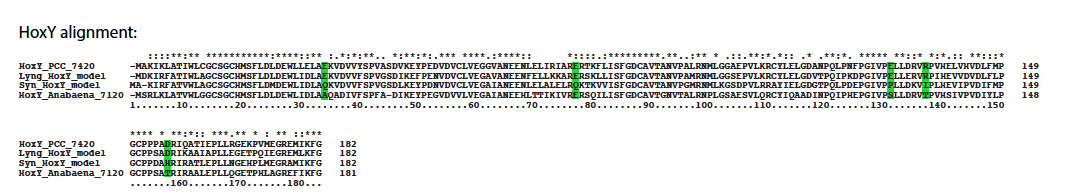


Supplementary Information 2: Multiple sequence alignment of hoxH and hoxY amino acid sequences from strains that exhibit Pattern 1 (*Anabaena variabilis* PCC 7120 and *Synechocystis* sp. PCC 6803) and Pattern 2 (*Microcoleus chthonoplaste*s PCC 7420 and *Lyngbya aestuarii* BL J) hydrogen production. The highlighted residues correspond to positions discussed in the main text as possible sites of significant variation, in the amino acid type, between the Pattern 1 and Pattern 2 while remaining conserved within the Pattern itself.
